# Antimicrobial Compounds from Leaf Extracts of *Jatropha curcas*, *Psidium guajava*, and *Andrographis paniculata*

**DOI:** 10.1155/2014/635240

**Published:** 2014-08-26

**Authors:** M. M. Rahman, S. H. Ahmad, M. T. M. Mohamed, M. Z. Ab Rahman

**Affiliations:** ^1^Department of Crop Science, Faculty of Agriculture, Universiti Putra Malaysia, 43400 Serdang, Selangor, Malaysia; ^2^Department of Chemistry, Faculty of Science, Universiti Putra Malaysia, 43400 Serdang, Selangor, Malaysia

## Abstract

The present research was conducted to discover antimicrobial compounds in methanolic leaf extracts of *Jatropha curcas* and *Andrographis paniculata* and ethanolic leaf extract of *Psidium guajava* and the effectiveness against microbes on flower preservative solution of cut Mokara Red orchid flowers was evaluated. The leaves were analyzed using gas chromatography-mass spectrometry. A total of nine, 66, and 29 compounds were identified in *J. curcas*, *P. guajava*, and *A. paniculata* leaf extracts, with five (88.18%), four (34.66%), and three (50.47%) having unique antimicrobial compounds, respectively. The experimental design on vase life was conducted using a completely randomized design with 10 replications. The flower vase life was about 6 days in the solution containing the *P. guajava* and *A. paniculata* leaf extracts at 15mg/L. Moreover, solution with leaf extracts of *A. paniculata* had the lowest bacterial count compared to *P. guajava* and *J. curcas*. Thus, these leaf extracts revealed the presence of relevant antimicrobial compounds. The leaf extracts have the potential as a cut flower solution to minimize microbial populations and extend flower vase life. However, the activities of specific antimicrobial compounds and double or triple combination leaf extracts to enhance the effectiveness to extend the vase life need to be tested.

## 1. Introduction

A major problem in Mokara Red orchid cut flowers is reduction in water uptake which could be due to the blockage of xylem vessels by microorganisms or air bubbles, thus, causing flower senescence and shortening of vase life [1, 2]. Floral senescence is an active process expressed as petal in-rolling, fading of colour, and wilting, caused by programmed cell death [3]. Besides, when the stem is cut, air is immediately aspired into all opened xylem conduits. This air will at first be restricted to the opened conduits. Since vase water bacteria cannot move from one xylem vessel to the other and polysaccharides excreted by bacteria move only partially up the stem, the blockage that occurs further up the stem is mainly due to air bubbles in the xylem conduits [4]. The orchids are an extremely diverse group, in terms of species numbers and also with respect to colour, shape, and smell. Although the pollination mechanisms of several species have been studied, deceptive systems still remain to be investigated [5]. Silver nitrate (AgNO_3_), 8-hydroxyquinoline citrate (8-HQC), sucrose, and citric acid (CA) have been used in vase solutions to extend the longevity of cut* Dendrobium* Pompadour flowers [6]. The sucrose acts as a food source, while CA (stabilizes pH to 3-4), AgNO_3_, and 8-HQC act as antimicrobial agents preventing the blockage of xylem vessels [7]. Currently, synthetic germicides containing AgNO_3_ are no longer used in commercial vase solutions because the silver can pollute the environment and cause damage to human health [8]. Thus, it is important to develop new substances for flower preservative solutions from sources of biological origin as alternative biocides for the floriculture industry.

Extraction is the basic step in the recovery and separation of bioactive antimicrobial compounds from plant resources before component analysis. The analysis and extraction of plant constituents are beneficial for enhancing, upgrading, and controlling the quality of natural product formulations. Plant derived metabolites have been valuable sources of different antimicrobial compounds used in the production of pharmaceuticals. These natural products, with antimicrobial and antibacterial properties, are reasonably harmless to man. These natural products can negate the expensive and insufficient supply of synthetic antimicrobial compounds.

*Jatropha curcas* is a physic nut belonging to the Euphorbiaceae family. In many subtropical and semiarid regions, it is traditionally used for its medicinal properties. The seeds contain semidry oil, which is useful for medicinal purposes. Leaf extracts of* J. curcas* had demonstrated insecticidal effects against crop pests such as* Helicoverpa armigera* and* Sitophilus zeamais* [9]. Siva et al. [10] had reported antifungal activities of crude medicinal plant extracts of 20 plant species, including* Ocimum sanctum*,* Ricinus communis*, and* J. curcas*. Besides, Rahman et al. [11] had also reported antifungal properties of* J. curcas* fruit. According to Kalimuthu et al. [12], the fungal pathogen growth of* Penicillium* sp. was inhibited by* J. curcas* extracts. These findings indicate that the extracts can control fungal diseases. Yin et al. [13] found that 9-hexadecenoic acid, a component present in* J. curcas* leaf extracts, has antifungal properties.

*Psidium guajava* L. is a widely cultivated crop belonging to the Myrtaceae family. Mature leaf extracts of* P. guajava* have been shown to contain antimicrobial properties [14]. The leaf extract of* P. guajava* contains phytochemicals such as flavonoid compounds (quercetin-3-O-α-L-arabinofuranoside, quercetin-3-O-*β*-D-arabinofuranoside, quercetin-3-O-*β*-D-glucoside, and quercetin-3-O-*β*-D-galactoside, quercetin-3-O-*β*-D-arabinofuranoside), which act as antimicrobials. The* P. guajava* leaf extract also contains squalene, which is commonly used in the cosmetic industry due to its antifungal properties [15, 16]. The phytol and bicycle[7.2.0]undec-4-ene,4,11,11-trimethyl-8-methylene-[1R-(1Rα,4Z,9S)] have antimicrobial [17, 18] activities while azulene has antifungal activities [19].

*Andrographis paniculata* belongs to the Acanthaceae family and is popularly known as king of bitters in English. It is an annual herbaceous plant which is commonly cultivated in Southern Asia, in China, and in some parts of South East Asia.* A. paniculata* has antimicrobial [20–22] and anti-snake-bite activities [23]. Diterpenoids and flavonoids are the main antimicrobial constituents of* A. paniculata* [24].

The leaves contain alkanes, flavonoids, and terpenoids. The bitter principle in the leaves is due to the presence of andrographolide lactone and kalmeghin. Four lactones, chuanxinlian A (deoxyandrographolide), B (andrographolide), C (neoandrographolide), and D (14-deoxy-11, 12-didehydroandrographolide), were isolated from the leaves [25]. Hence, the leaf extract of* A. paniculata* is currently being used as a natural biocide due to the antimicrobial compounds.

Currently, there is a growing public concern on the possible risks of using synthetic fungicides on human health and environment [26]. Therefore, there is a need for alternative antimicrobial agents of biological origin that would not have adverse effects on the environment and human health. Information on the effect of extracts of* J. curcas*,* P. guajava*, and* A. paniculata* leaves as a biopreservative agent against microbes in vase solutions of Mokara Red orchid cut flowers is still scarce. Hence, this study was conducted to identify presence of natural antimicrobial compounds in* J. curcas*,* P. guajava*, and* A. paniculata* leaves extracts due to the* J. curcas*,* P. guajava*, and* A. paniculata* leaves extracts prospective as a cut flower preservative solution to reduce microbial populations and extend the flower vase life.

## 2. Materials and Methods

### 2.1. Leaf Extracts and Detection of Antimicrobial Compounds

Leaves of* J. curcas*,* P. guajava*, and* A. paniculata* were collected from the University Agriculture Park, Universiti Putra Malaysia (UPM), Serdang, Selangor. Insect- and disease-free leaves of* J. curcas* and* P. guajava* from nodes seven to eight below the youngest leaves were collected at random from 25 plants, while 17-week-old* A. paniculata* plants were harvested at 10 cm above the soil surface and the leaves from 50 plants were used for the extraction.

The collected leaves were processed in the Postharvest Laboratory, Department of Crop Science, Faculty of Agriculture, UPM. The leaves were extracted according to the method of Rahman et al. [11], with some modifications. The samples were washed with distilled water and air-dried at ambient temperature (25 ± 2°C) to remove excess water and then oven-dried (Memmert, ULM 500, Germany) at 45°C until a constant moisture of 14% was obtained. Then the leaves were ground separately in a grinding mill (POLYMIX System MFC 13 CZ, the Netherlands). Twenty-gram crushed sample was placed into a 500 mL Erlenmeyer flask.* J. curcas* and* A. paniculata* crushed samples were soaked in 120 mL methanol solvent, while the* P. guajava* sample was soaked in 120 mL ethanol. Each flask was covered with a piece of an aluminium foil and then sealed with parafilm tape to prevent contamination and evaporation of the solutions. The suspended solutions were left to stand for 7 days, after which the solutions were filtered (filter paper 90 mm, Toyo Roshi Kaisha, Ltd., Japan) and evaporated under vacuum using a rotary evaporator (Model CA-1310 Eyela, Tokyo Rikakikal Co., Ltd., Japan) to remove the solvent.

Crude leaf extracts of* J. curcas*,* P. guajava*, and* A. paniculata* were analyzed quantitatively using a gas chromatography-mass spectrometry (GC-MS) (GC-17A/GCMS-QP5050A, Shimadzu, Japan) equipped with DB-wax fused silica capillary column (30 m × 0.25 mm i.d., 0.5 *μ*m film thickness). The method was based on Hashimoto et al. [27] with some modifications. Helium was used as the carrier gas at a constant flow rate of 1 mL/min. The injector and interface temperatures were 250 and 260°C, respectively, and solvent delay was 4 min. The column temperature was raised from 40 to 240°C (at 5°C/min) and finally to 270°C (at 10°C/min). Initial oven temperature was 50°C for 3 min, then increased to 250°C at a rate of 5°C/min, and then held at 250°C for 15 min. The* J. curcas* and* A. paniculata* leaf extracts were diluted in methanol (1/100, v/v), while* P. guajava* leaf extracts were diluted in ethanol (1/100, v/v). Then 1 *μ*L of the diluted extract was manually injected into the GC-MS in splitless mode. Electron ionization system with ionization energy of 70 eV and 50–500* m/z* scanning range was used for detecting the antimicrobial compounds. Identification of the antimicrobial chemical compounds was based on comparison of retention times and computer matching of the mass spectra with those of the National Institute of Standards and Technology (NIST 08 and NIST 08s) library and by direct comparison with published data. Leaf extract constituents were expressed as percentage peak area normalization.

### 2.2. Determination of Vase Life, pH, and Microbial Populations

Mokara Red orchid cut flowers were obtained from a commercial farm in Selangor, Malaysia. Flowers with 75% opened florets and sufficiently strong stems were harvested by cutting the stem 45–50 cm in length, between 7 and 8 a.m. The flowers were transported within 1 h after harvest to the Postharvest Laboratory, Department of Crop Science, UPM, for further treatment.

The basal stem of each flower was recut (5 cm) under deionized water to avoid stem-end air emboli. Forty-millilitre flower preservative solution containing 2% sucrose and 3% CA was placed in a 50 mL glass centrifuge tube (DURAN centrifuge tubes, SCHOTT North America, Inc. NY 10523 USA). Then each flower stem was placed in the tube with the stem-end of each flower being held straight, 1 cm from the base of the tube, with the support of a cotton wool plugged around the stem at the tube's rim to prevent evaporation of the preservative solution. Ten tubes of flowers were prepared with each flower per tube representing one replication. The flowers were kept at ambient temperature (22 ± 2°C) and under continuous white fluorescent light (1.2 klux) throughout the vase life. The preservative solutions were not renewed until the end of the vase life. Vase life ended when 30% of flowers appeared unattractive due to bud that remains closed and wilted, petal discoloration and wilting, floret epinasty and drop, and stem yellowing. The pHs of the preservative solutions were determined with a pH meter (Model GLP-21; CRISON; Barcelona) at the end of the flower vase life.

Microbial populations of the preservative solutions were determined according to the method of Rahman et al. [11], with some modifications. For the microbial population determination (bacteria count and fungal growth) only the 15 mg/L preservative solutions containing leaf extracts of* J. curcas*,* P. guajava*, and* A. paniculata*, from the vase life study, were used. At the end of the flower vase life, 1 mL of preservative solution from the centrifuge tube was sampled into a universal bottle (100 mL) containing 9 mL of distilled water. This was used as the vase stock solution for further dilution. Then, 1 mL of the stock solution was added to 9 mL of distilled water. Each step resulted in a further 8-fold and 6-fold change, from the previous diluted preservative solution, for bacterial count and fungal growth determinations, respectively. The samples were mixed and homogenized using a vortex mixer (Model SA-8; STUART; Switzerland), at a speed of 2500 rpm for 2 min.

Nutrient agar (NA) and potato dextrose agar (PDA) were prepared according to the manufacturer's instruction, as labelled on the bottle. Fourteen grams of NA and 20 g of PDA were diluted separately in 1 L distilled water and the solutions were boiled for 1 min, autoclaved at 121°C and 15 psi for 15 min, and cooled. Twenty five mL pre-autoclaved NA and PDA was poured into each 90 mm diameter sterile petri dish. The agars were allowed to solidify at room temperature. Then, 100 *μ*L of 8-fold and 6-fold of the final diluted vase solutions was plated onto the NA and PDA petri dishes, respectively. After the plating process, 1 *μ*L of the diluted* J. curcas*,* P. guajava*, and* A. paniculata* pure leaf extracts (using the same dilution as the GC-MS injection) was added into the NA and PDA petri dishes. The inoculated petri dishes containing NA and PDA were incubated upside down in the incubator for 2 and 7 days at 28 ± 2°C, respectively. Bacterial counts were determined by the standard plate counting method (by counting the number of colonies formed after incubation) to get the number of colony forming units/mL (cfu/mL) [28]. The diameter (mm) of fungus growth on PDA medium was measured with a millimeter ruler.

#### 2.2.1. Experimental Design and Statistical Analysis

The experiments on vase life, pH, and microbial populations were conducted using the completely randomized design with five treatments comprising three leaf extracts (*J. curcas*,* P. guajava*, and* A. paniculata*) and two controls (tap water and 8-HQC), replicated 10 times. Additionally, each stock dilution (in the petri dishes) was repeated five times (five petri dishes/treatment). All the collected data were subjected to an analysis of variance (ANOVA) to define the differences between treatments. The treatment means were compared using Duncan's multiple range test (DMRT) at *P* ≤ 0.05 (SAS Software Version 9.1).

## 3. Results and Discussion

### 3.1. Antimicrobial Compounds in Leaves Extracts

**Figure 1:**
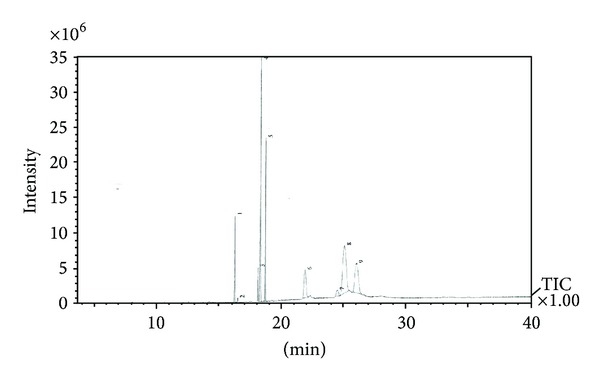
A gas chromatography-mass spectrometry chromatogram peak profile of* Jatropha curcas* leaf extract.

Nine peaks were detected in the GC-MS chromatograms of* J. curcas* methanol leaf extracts (Figure 1). There were five antimicrobial compounds that were present in considerable amounts (88.18%), and another four antimicrobial compounds comprised 11.82% (Table 1). A component 9-hexadecenoic acid [13] present in* J. curcas* leaf extracts has antimicrobial properties. Compounds 10-octadecenoic acid methyl ester [29], 9,12-octadecadienoic acid (Z,Z)-, 9,12-octadecadienoic acid methyl ester [30], and n-hexadecenoic acid [31] have been reported to have antimicrobial activity. The presence of these compounds makes* J. curcas* leaves a source of bioactive compounds. Other minor compounds like hexadecenoic acid methyl ester [30] have antibacterial properties; both 9-hexadecenoic acid methyl ester (Z)- and octadecanoic acid methyl ester [32] have antioxidant activities, whereas octadecanoic acid [33] has antimicrobial activity. All these compounds are bioactive compounds. The retention times and antimicrobial compounds detected in the methanol extracts of* J. curcas* leaves are presented in Table 1.

**Figure 2:**
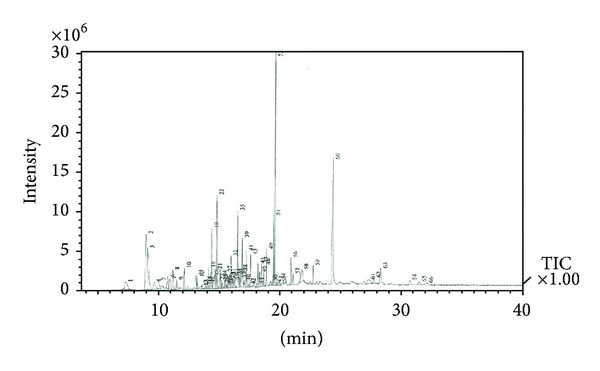
Gas chromatography-mass spectrometry profile of* Psidium guajava* leaf extract.

A total of 66 peaks from ethanolic leaf extract of* P. guajava* were detected in the GC-MS chromatograms (Figure 2). The results showed that four antimicrobial compounds were present as major antimicrobial compounds (34.66%) and another 25 (46.86%) were moderately active antimicrobial compounds (Table 2). Squalene has been indicated to contain antifungal properties and is commonly used in the cosmetic industries [15, 16]. Phytol has antimicrobial and antioxidant activities [17], bicyclo[7.2.0]undec-4-ene,4,11,11-trimethyl-8-methylene-,[1R-(1Rα,4Z,9S)] molecule has antimicrobial properties [18], and azulene has antifungal activities [19]. All these compounds were present in the* P. guajava* leaves which were proven by previous research [15–19]. In the present study, the* P. guajava* leaf extracts had four most abundant antimicrobial components including 11.63% of squalene, 10.30% of phytol, 7.54% of bicyclo[7.2.0]undec-4-ene,4,11,11-trimethyl-8-methylene-,[1R-(1Rα,4Z,9S)], and 5.19% of azulene (Table 2). The medicinal activity of* P. guajava* leaf is attributed to the antimicrobial compounds present in the leaf extracts. The retention times and antimicrobial compounds detected in the ethanol extracts of* P. guajava* leaves are presented in Table 2.

**Figure 3:**
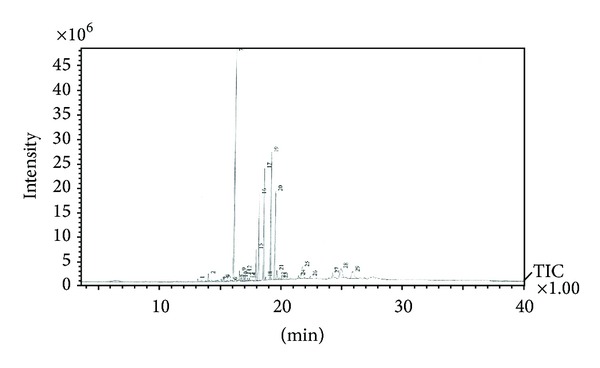
Gas chromatography-mass spectrometry profile of* Andrographis paniculata* leaf extract.

The* A. paniculata* leaf extracts in methanol showed 29 peaks as identified from the GC-MS chromatogram (Figure 3). There were three major (50.47%) antimicrobial compounds (Table 3). These include 24.64% of hexadecenoic acid methyl ester, 13.65% of 9,12,15-octadecatrienoic acid methyl ester (Z,Z,Z)-, and 12.18% of 9,12-octadecadienoic acid methyl ester. There were nine (38.30%) moderately active antimicrobial compounds: 9.92% of phytol, 9.75% of 10-octadecenoic acid methyl ester, 5.46% of n-hexadecenoic, 5.27% of 9-hexadecenoic acid, 3.48% of octadecanoic acid methyl ester, 1.22% of heptadecanoic acid methyl ester, 1.14% of eicosanoic acid methyl ester, 1.04% of 9-hexadecenoic acid methyl ester (Z)-, and 1.02% of squalene. In addition there were 17 minor (11.23%) antimicrobial compounds (Table 1). The antimicrobial compounds in the* A. paniculata* leaf extracts contributed to its medicinal activity. The retention times and the antimicrobial compounds present in* A. paniculata* leaf extracts are presented in Table 3.

### 3.2. Flower Vase Life, pH, and Microbial Populations

Flowers in tap water (control) had significantly shorter (50%) vase life compared to flowers in preservative solution with 8-HQC (chemical germicide) (Table 4). However, flowers in tap water had similar vase life as flowers in other treatments except those flowers in preservative solutions containing 15 mg/L* P. guajava* or* A. paniculata* leaf extracts, whereby these flowers had 2-day longer vase life compared to flowers in the tap water.

The final pH of the preservative solution was determined after the end of flower vase life. The pH of tap water at the end of vase life was significantly higher (50%) compared to the final pH of preservative solution treated with 8-HQC (Table 4). The final pH of preservative solutions with tap water was similar to the other treatments except for the pH of preservative solutions containing 15 mg/L* P. guajava* or* A. paniculata* leaf extracts. The pH of these preservative solutions containing 15 mg/L* P. guajava* or* A. paniculata* leaf extracts was lower (32–34%) than the final pH of tap water. The increase of pH could be due to the increase in OH^−^ group resulting from metabolic activities in the flowers [34]. There was absence of bacteria in the 8-HQC preservative solution and the flower vase life was 8 days (Table 5). The* A. paniculata* leaf extract preservative solution had the lowest bacterial count followed by* P. guajava* and* J. curcas*, and the flower vase life was about 6 days. The 8-HQC totally inhibited fungal growth in the preservative solution (Table 5). Preservative solution treated with* J. curcas* had significantly lower fungal growth than preservative solution treated with* P. guajava* and* A. paniculata*. However, the effects of these fungi to flower life were found to be less damaging than the effects of bacteria. Commonly, tap water alone is used to preserve cut flowers in a vase, resulting in a shorter vase life compared to using preservative solutions into which sugar has been added as a food source. The bacteria are attracted to the sugar in the vase solution and their growth blocks xylem vessels of the cut flower stem, thus preventing water uptake by the flowers. The vase life of cut flowers in preservative solutions containing 15 mg leaf extracts/L of either* P. guajava* or* A. paniculata* was extended by 2 days compared to vase life of flowers in tap water. This could be due to the antimicrobial compounds identified in the leaf extracts. In* P. guajava*, the antimicrobial compounds were squalene, phytol, bicyclo[7.2.0]undec-4-ene,4,11,11-trimethyl-8-methylene-,[1R-(1Rα,4Z,9S)], and azulene, whilst the antimicrobial compounds in* A. paniculata* were hexadecenoic acid methyl ester, 9,12,15-octadecatrienoic acid methyl ester (Z,Z,Z)-, and 9,12-octadecadienoic acid methyl ester. Thus, the 15 mg leaf extracts/L could substitute for 8-HQC, since 8-HQC is a chemical germicide that is not easily available. Furthermore it is expensive and could pollute the environment. To be effective in extending vase life, the leaf extracts need to be used in proper concentrations. However, if the concentration is too low or too high, then it might not be effective or could even cause an antagonistic effect on vase life. Possibly, the leaf extract could be used in double or triple combination to enhance each of their effectiveness to extend the vase life. The same antimicrobial compounds have been identified in the leaf extracts of* P. guajava* and* A. paniculata* [15–19, 29–31, 35]. Adding a suitable natural biocide into the preservative solution could inhibit the development of microbes and extend vase life of cut flowers.

## 4. Conclusion 

In the present study, the leaf extracts from* J. curcas*,* P. guajava*, and* A. paniculata* were found to possess antimicrobial activities. The antimicrobial activity of* P. guajava* and* A. paniculata* leaf extracts appeared to be responsible for extending the cut flower vase life by 2 days. Both the 15 mg leaf extracts/L of* P. guajava* and* A. paniculata* were able to control the microbes in the preservative solution (petri dishes), thus extending the flower vase life by reducing the pH of the preservative solution. However, further studies on the specific antimicrobial compound activities need to be tested and, possibly, a double or triple combination of the leaf extracts could enhance each of their potencies. Thus, these leaf extracts could be used as natural biocides to control microbes and extend the cut flower vase life.

## Figures and Tables

**Table 1:** Antimicrobial compounds, retention times, and percentages of methanol leaf extract of *Jatropha curcas* analyzed by a gas chromatography-mass spectrometry.

Number	R. time	Compound name	(%)
1	16.14	Hexadecanoic acid methyl ester (palmitic acid methyl ester)	6.51
2	16.39	9-Hexadecenoic acid methyl ester (Z)-(palmitoleic acid methyl ester)	0.33
3	17.99	Octadecanoic acid methyl ester (stearic acid methyl ester)	2.87
4	18.18	10-Octadecenoic acid methyl ester (stearic acid methyl ester)	20.04
5	18.59	9,12-Octadecadienoic acid methyl ester (linoleic acid methyl ester)	12.43
6	21.81	n-Hexadecanoic acid (palmitic acid)	10.10
7	24.44	Octadecanoic acid (stearic acid)	2.11
8	24.99	9-Hexadecenoic acid (palmitoleic acid)	28.31
9	25.95	9,12-Octadecadienoic acid (Z,Z)-(linoleic acid)	17.30

**Table 2:** Antimicrobial compounds, retention times, and percentages of methanol leaf extract of *Psidium guajava* analyzed by a gas chromatography-mass spectrometry.

Number	RT	Compound name	(%)
60	24.27	Squalene	11.63
52	19.49	Phytol	10.30
2	8.98	Bicyclo[7.2.0]undec-4-ene, 4,11,11-trimethyl-8-methylene-,[1R-(1Rα,4Z,9S)]	7.54
3	9.10	Azulene, 1,2,3,3A,4,5,6,7-octahydro-1,4-dimethyl-7-(1-methylethenyl)-, [1R-(1)]	5.19
22	14.73	Globulol	4.35
35	16.44	Hexadecenoic acid ethyl ester	3.58
19	14.32	1,6,10-Dodecatrien-3-ol, 3,7,11-trimethyl-, (Z)-	3.32
51	19.37	9,12,15-Octadecatrienoic acid methyl ester (Z,Z,Z)-	3.25
39	16.82	Tetracyclo[6.3.2.0(2,5).0(1,8)]tridecan-9-ol, 4,4-dimethyl-	3.02
58	21.73	n-Hexadecanoic acid	2.12
1	7.33	Copaene	1.97
8	11.15	Naphthalene, 1,2,4a,5,8,8a-hexahydro-4,7-dimethyl-1-(1-methylethyl), [1S-(1)]	1.96
41	17.16	Isoaromadendrene	1.93
63	28.01	Benzene	1.65
10	12.10	Naphthalene	1.64
49	18.82	Linoleic acid ethyl ester	1.60
43	17.51	Isoaromadendrene epoxide	1.48
47	18.43	Ethyl 9-octadecenoate (E)-	1.45
20	14.55	Cubenol	1.40
32	15.90	1-Naphthalenol, 1,2,3,4,4a,7,8,8a-octahydro-1,6-dimethyl-4-(1-methylethyl)-	1.38
56	20.83	4-Isopropenyl-4, 7-dimethyl-1-oxaspiro[2.5]octane	1.33
7	10.91	Cyclohexane, 1-ethenyl-1-methyl-2-(1-methylethenyl)-4-(1-methylethylidene)-	1.24
6	10.74	1H-Benzocycloheptene, 2,4a,5,6,7,8,9,9a-octahydro-3,5,5-trimethyl-9-methyle	1.23
61	27.19	9,12,15-Octadecatrienoic acid methyl ester (Z,Z,Z)-	1.19
18	14.08	Epiglobulol	1.15
59	22.66	4,4,8- Trimethylbicyclo [6.3.1.0(1,5)]dodecane-2,9-diol	1.11
45	18.12	Cyclopropanecarboxylic acid, 2,2-dimethyl-3(Z)-[.alpha.-(carboxymethyl)-ally]	1.10
4	9.65	Naphthalene1,2,3,4,4a,5,6,8a-octahydro-7-methyl-4-methylene-1-(1-methylet)-	1.09
5	10.01	.alpha.-Caryophyllene	1.01

**Table 3:** Antimicrobial compounds, retention times, and percentages of methanol leaf extract of *Andrographis paniculata* analyzed by a gas chromatography mass spectrometry.

Number	RT	Compound name	(%)
7	16.11	Hexadecanoic acid methyl ester (palmitic acid methyl ester)	24.64
9	16.54	9-Hexadecenoic acid methyl ester (Z)-(palmitoleic acid methyl ester)	1.04
12	17.04	Heptadecanoic acid methyl ester	1.22
15	17.96	Octadecanoic acid methyl ester (stearic acid methyl ester)	3.48
16	18.14	10-Octadecenoic acid methyl ester (stearic acid methyl ester)	9.75
17	18.55	9,12-Octadecadienoic acid methyl ester (linoleic acid methyl ester)	12.18
19	19.11	9,12,15-Octadecatrienoic acid methyl ester (Z,Z,Z)-	13.65
20	19.49	Phytol	9.92
21	19.68	Eicosanoic acid methyl ester	1.14
25	21.80	n-Hexadecanoic acid	5.46
27	24.27	Squalene	1.02
28	24.99	9-Hexadecenoic acid	5.27

**Table 4:** Vase life of cut Mokara Red orchid flower and pH (final) of the flower preservative solutions as affected by *Jatropha curcas*, *Psidium guajava,* and *Andrographis paniculata* leaf extracts, tap water, and 8-hydroxyquinoline citrate (8-HQC).

Treatment	Concentration (mg/L)	Vase life (day)	pH
Tap water (control)	—	4.26 ± 0.26e^z^	8.70 ± 0.03a

8-HQC (control)	125	8.61 ± 0.11a	4.04 ± 0.01g

*J. curcas *	5	4.18 ± 0.16e	8.03 ± 0.02b
	10	5.07 ± 0.20d	7.47 ± 0.02c
	15	5.63 ± 0.19cd	6.35 ± 0.03e
	20	3.59 ± 0.21f	8.64 ± 0.04a

*P. guajava *	5	4.43 ± 0.20e	7.04 ± 0.04d
	10	5.35 ± 0.20d	6.60 ± 0.03e
	15	6.38 ± 0.21b	5.73 ± 0.02f
	20	3.98 ± 0.21ef	7.60 ± 0.03c

*A. paniculata *	5	4.30 ± 0.30e	7.32 ± 0.02cd
	10	5.14 ± 0.32d	6.65 ± 0.02e
	15	6.02 ± 0.30bc	5.84 ± 0.03f
	20	3.91 ± 0.34ef	7.70 ± 0.04bc

2% sucrose and 3% citric acid were added to each flower preservative solution.

Vase life ended when 30% of flowers appeared unattractive due to bud that remains closed and wilted, petal discoloration and wilting, floret epinasty and drop, and stem yellowing. Data are means ± SE; *n* = 10.

^z^Means followed by the same letter within each column are not significantly different by DMRT (*P* ≤ 0.05).

**Table 5:** Bacterial count and fungal growth at the end of vase life of Mokara Red orchid flowers in petri plates containing the flower preservative solution as affected by leaf extracts of *Jatropha curcas*, *Psidium guajava*, and *Andrographis paniculata* and 8-hydroxyquinoline citrate (8-HQC).

Treatment	Bacterial count	Fungal growth
	(cfu 10^8^ mL^−1^)^z^	Diameter (mm)
Control, 8-HQC (125 mg/L)	None	None
*J. curcas* (15 mg/L)	158.00 ± 2.41a	18.45 ± 1.75b
*P. guajava* (15 mg/L)	75.00 ± 2.59b	25.25 ± 1.33a
*A. paniculata* (15 mg/L)	37.00 ± 1.64c	27.23 ± 1.49a

2% sucrose and 3% citric acid were added to each flower preservative solution. Data are means ± SE; *n* = 10.

Vase life ended when 30% of flowers appeared unattractive due to bud that remains closed and wilted, petal discoloration and wilting, floret epinasty and drop, and stem yellowing.

^z^Means followed by the same letter within each column are not significantly different by DMRT (*P* ≤ 0.05).

## Abbreviations

8-HQC: 8-Hydroxyquinoline citrate
AgNO_3_: Silver nitrate
CA: Citric acid
cfu: Colony forming units
GC-MS: Gas chromatography-mass spectrometry.
